# Witnessing hateful people in pain modulates brain activity in regions associated with physical pain and reward

**DOI:** 10.3389/fpsyg.2013.00772

**Published:** 2013-10-23

**Authors:** Glenn R. Fox, Mona Sobhani, Lisa Aziz-Zadeh

**Affiliations:** ^1^Neuroscience Graduate Program, University of Southern CaliforniaLos Angeles, CA, USA; ^2^Brain and Creativity InstituteLos Angeles, CA, USA; ^3^Division of Occupational Science and Occupational TherapyLos Angeles, CA, USA

**Keywords:** empathy, observation of pain, social group membership, fMRI, pain matrix

## Abstract

How does witnessing a hateful person in pain compare to witnessing a likable person in pain? The current study compared the brain bases for how we perceive likable people in pain with those of viewing hateful people in pain. While social bonds are built through sharing the plight and pain of others in the name of empathy, viewing a hateful person in pain also has many potential ramifications. In this functional Magnetic Resonance Imaging (fMRI) study, Caucasian Jewish male participants viewed videos of (1) disliked, hateful, anti-Semitic individuals, and (2) liked, non-hateful, tolerant individuals in pain. The results showed that, compared with viewing liked people, viewing hateful people in pain elicited increased responses in regions associated with observation of physical pain (the insular cortex, the anterior cingulate cortex (ACC), and the somatosensory cortex), reward processing (the striatum), and frontal regions associated with emotion regulation. Functional connectivity analyses revealed connections between seed regions in the left ACC and right insular cortex with reward regions, the amygdala, and frontal regions associated with emotion regulation. These data indicate that regions of the brain active while viewing someone in pain may be more active in response to the danger or threat posed by witnessing the pain of a hateful individual more so than the desire to empathize with a likable person's pain.

## Introduction

When we watch a film that involves the downfall of a dangerous antagonist, the moment of his demise draws our focus to the pain he experiences as a result of his behavior. As viewers, we watch carefully to determine whether his demise is permanent, as this is critical for predicting his potential for retribution in the future. Conversely, witnessing the suffering of a protagonist elicits empathy for his suffering, and perhaps a shared awareness of his physical pain. Comparing these experiences, we can wonder which experience will draw a greater response at the level of the brain. Will regions of the brain that respond to pain in others be more active for witnessing a hateful person as a result of the heightened awareness of his pain? Or will the same pain processing regions be more active in response to witnessing a likable person's pain as a result of the desire to empathize with him?

Previous studies examining the observation of pain have found activity in a network of brain regions often called the “pain matrix” which includes the insula cortex, the anterior cingulate cortex (ACC), and the somatosensory cortices (Singer et al., 2004; Avenanti et al., 2005; Jackson et al., 2006; Lamm et al., 2007). Note that brain activity in these regions is found in a broad array of experimental paradigms, so they are not uniquely tuned to pain *per se*; we therefore use the term “pain matrix” loosely, and for the sake of brevity to refer to the regions mentioned above (Please see Supplementary Figure 1 for a broad summary of other commonly cited roles of these regions). Subsequent studies have shown that activity in these regions is modulated by the relationship between the viewer and the person in pain. For example, a previous functional Magnetic Resonance Imaging (fMRI) study considered whether activity in the pain matrix is modulated by racial group membership (Xu et al., 2009). In this study, Caucasian and Chinese subjects viewed pained expressions on the faces of racial in-group and out-group members as they received injections to the cheek. Observation of in-group members experiencing pain was associated with increased activity in the ACC and the insula compared with viewing out-group members receiving the same stimulus. This study suggests that perception of these racial categories modulates activity in brain regions associated with pain processing, and may be related to our increased empathy and sensitivity to viewing in-group members in pain (Xu et al., 2009). In addition to race, other factors have also been found to modulate activity in the pain matrix, such as: the perceived fairness of others (Singer et al., 2006), experience with the type of painful stimulus, e.g., acupuncturists viewing needle injections (Cheng et al., 2007), interpersonal love (Chen et al., 2010), viewing individuals who experience pain differently than ourselves (Lamm et al., 2010), or different social contexts (Akitsuki and Decety, 2009).

The findings from studies of observation of pain and group membership have found that the regions of the pain matrix are likely to be more active when viewing in-group, liked, and similar people compared with viewing out-group, disliked, or dissimilar people in pain (Singer et al., 2004, 2006; Lamm and Decety, 2008; Han et al., 2009; Lamm et al., 2010, 2011). Given that regions involved in self-pain processing are also active when viewing in-group members in pain, and their levels of activity have been correlated to individual differences in empathy (Singer et al., 2004), activity in regions of the pain matrix is also often referred to as being “empathy-related” (Singer et al., 2006).

There are, however, exceptions to the in-group bias model for activity in the regions of the pain matrix. It has been found that for groups sharing strong, ongoing cultural conflict, witnessing a member of one's in-group in physical pain does not necessarily elicit greater activity in the pain matrix when compared with witnessing an out-group member, and activity in the pain matrix was found to be highest when witnessing a non-conflicting out-group member in pain (Bruneau et al., 2012). This finding does not directly contradict the in-group bias *per se*, but it does present an exception, as the brain activity for in-group members' pain was not greater than the adversarial out-group.

Relatedly, one study found that perceiving a hated person's face, compared with that of a neutral person, elicited increased activity in regions of the brain that closely overlap with regions of the pain matrix, and activity in these regions was correlated to the subjective rating of hate participants felt for the hated people (Zeki and Romaya, 2008). Although this study does not focus on witnessing people in pain, it raises the question of whether activity in these regions is specific to understanding others' pain, or can be elicited by conditions that are inherently more emotionally salient than the other conditions in a given experiment.

The role of attention in modulating activity in the pain matrix has also been studied more directly. Gu and Han (2007a) found that the regions of the pain matrix are sensitive to top down modulation. In this study, participants viewed images of depicted pain and were asked to focus on the pain intensity or to count the number of hands present in the image. Their findings showed greater activity in the pain matrix regions for conditions in which participants focused on the pain of the actors in the image, suggesting that the pain-related content of an image is not automatically linked to increases in pain-related regions, but the type of focus brought to an image plays a role in how the content of the image is processed at the level of the brain.

The concert of brain regions associated with viewing a disliked person in pain has also been shown to include regions associated with feeling pleasure. For instance, a recent brain imaging study showed that viewing fans of a rival soccer team in painful situations elicits activity in the nucleus accumbens, part of the brain's dopamine reward pathway (Hein et al., 2010). Other studies have also indicated that one may feel reward at watching rivals suffering-otherwise known as schadenfreude (Singer et al., 2006; Takahashi et al., 2009; Dvash et al., 2010; Cikara et al., 2011). Taken together, these findings suggest that activity in the striatum, which includes the nucleus accumbens in its ventral regions, and the caudate and putamen in its dorsal regions, is associated with the feeling of pleasure derived from watching a disliked person suffering.

In the current study, we sought to determine how viewing hateful people in pain modulates brain activity in comparison to viewing likable people in pain. To address this question, 19 right-handed Jewish males viewed two categories of people receiving a painful injection: (1) likeable, open-minded individuals; and (2) hateful, dangerous, and disliked, anti-semitic neo-Nazi individuals. We chose to use these social groups because, unlike other studies on race, there is no social stigma associated with Jewish individuals disliking individuals from neo-Nazi backgrounds and there is a one-sided danger posed by the anti-Semitic group. Thus, this provides a situation where the responses are not dampened by the process of trying to cognitively subdue a stigmatized response, which may be the case when white participants view individuals from other races (Cunningham et al., 2004).

This design allows us to further elucidate the circumstances that modulate activity in pain processing regions. Previous reports have shown that brain activity in the pain matrix is modulated by others who are from out-groups (Bruneau et al., 2012), who are disliked (Singer et al., 2006), and dissimilar (Lamm et al., 2011), but it remains to be seen how we treat the pain of someone who hates us and also poses a potential danger—to our body and to our ideology. This creates a natural question to be addressed using fMRI. For instance, insofar as brain processes, hateful people could be treated similarly to disliked, unfair people as in the previous studies, and the response to their pain could be dampened by the participant's lack of willingness to share the experience with the hateful person in pain. In this framing, the in-group bias for these regions would “win,” and drive up the responses for viewing the likable people in pain. Conversely, witnessing hateful people in pain might elicit an increase in the level of activity in the regions of the pain matrix due to the arousing nature of the experience. The potential threat that the hateful person elicits may accentuate the neural response as well.

We predicted, based on the evidence favoring the in-group bias (Singer et al., 2006) that regions associated with pain processing (the ACC, insula, and somatosensory cortices) would show greater levels of activity for witnessing likable people in pain. Also in line with previous results, we expected to find increased activity in the striatum when subjects viewed hateful people experiencing pain (Takahashi et al., 2009; Dvash et al., 2010; Cikara et al., 2011).

In addition, methods for measuring “functional connectivity” can be used to examine how other brain regions may modulate the regions active while observing people in pain. Previous studies on pain and emotion regulation have shown robust functional connections between components of the pain matrix, such as the ACC and the insula, with regions involved with emotion regulation, fear, and basic emotion (Bantick et al., 2002; Bishop et al., 2004a,b; Ochsner and Gross, 2005; Banks et al., 2007; Zaki et al., 2007; Benuzzi et al., 2009; Yoshino et al., 2010; Kanske and Kotz, 2011; Lamm et al., 2011). Based upon these studies, we hypothesized that activity in the ACC and the insula, as elicited by performing whole brain contrasts between viewing hateful vs. likable people in pain, would show connectivity with medial prefrontal regions, other emotion-related brain regions such as the amygdala, as well as with reward-associated regions in the striatum.

## Materials and methods

### General design

Nineteen right-handed Jewish males were enrolled in our study. Each participant began the session with a 1-h training session where he learned about eight stories of people (hereafter referred to as protagonists or targets)-half of whom were hateful, and held strongly anti-Semitic beliefs, and half of whom were likable and held tolerant beliefs. Participants were asked after each story to rate the target along a number of dimensions in order to form an index of how much the participant liked the target. After the training session and a break, participants were placed in the scanner where they saw the same targets receiving a painful injection (the pain condition), or a touch from a q-tip (the control condition). After the scan session, participants were given a series of questionnaires and an interview and were debriefed as to the study's goals. Interviews followed a script to identify how the participants felt about the targets when they viewed them in the scanner in addition to how well they remembered the targets. All subjects provided informed consent and all research was conducted in accordance with U.S.C.'s institutional review board policies concerning research involving human subjects.

### Participants

Nineteen Jewish males (18–30 years old, mean = 22, *SD* = 3.5) participated in our study. We chose to include only male participants based upon a previous result that showed that male subjects' brain activity while viewing others in pain was more differentially modulated by fairness and liking than in female subjects (Singer et al., 2006). All participants scored highly (mean = 42.4/48, *SD* = 5.6) on a self-rating measure of Jewish identity (Phinney, 1992), as well as on a modified scale (Schmitt et al., 2002) that asked subjects to rate how closely they felt affiliated with the Jewish religion on an eight-point Likert scale (mean = 39/48, *SD* = 3.8). Three subjects had to be removed from the analysis, two due to technical problems involving the scanner, and one for recognizing one of the actors in the stimuli, reducing the final number to 16 subjects.

### Pre-scan training

The experimental session was broken into two parts: a training session and a scanning session. We modeled our approach after a previous study on social emotions, which also consisted of an in-depth training session followed by a scanning session (Immordino-Yang et al., 2009). The goal of this approach was to allow participants to build a rich emotional understanding of the images and stories.

In the training session, participants were introduced to eight protagonists through the use of “mini-documentaries” depicting stories and photos from the protagonist's life history. The stories were created by searching for biographies and testimonials found in various media, and combining facts and quotes from these works into profiles that were one thousand words in length. Four of the stories involved protagonists from positive, uplifting and accepting backgrounds (likable targets), the other four stories involved protagonists from negative, ungrateful and closed-minded backgrounds (hateful targets). To account for possible sex differences, in each condition (likable targets; hateful targets) there were two male and two female protagonists. The stories were designed primarily to induce feelings of threat, danger and dislike for the hateful targets, and tolerance, safety and liking toward the likable targets. As such, we used findings from social psychology studies on liking behavior to manipulate the likableness of the targets (Aronson and Linder, 1965; Pratkanis and Aronson, 2001; Aronson, 2004). Likable targets were characterized as being open-minded, intelligent, grateful, and positive in nature. By contrast, hateful targets were strongly racist and anti-semitic, at times violent, uninterested in education, cynical of the world and expressly ungrateful for gifts bestowed to them. Stories were constructed to have parallel structure and elements. For instance, each story detailed events from childhood that shaped the belief system of the protagonist, and described role models that shaped the protagonist's behavior. The targets in the movies were played by actors randomly placed in the hateful or likable conditions to control for physical appearance. Samples of these vignettes can be found in the Supplementary Material; all stories and constructs were piloted for validity and reliability prior to the study.

After each story, participants filled out a brief questionnaire regarding how much they liked the target, how much time they would like to spend with the target and how much they thought the target would like them. The questionnaire consisted of an open-ended question asking participants to describe what stood out to them about the story, as well as eight items asking subjects to rate the target on a Likert scale ranging from one to five, with five being the most likable. Subjects' ratings on individual items were then summed to give an aggregate score for each target, allowing an available range of scores from 8 to 40, with 8 being strongly hateful or disliked, and 40 being extremely likable. This questionnaire is included in the Supplementary Material. At the end of the pre-scan session, as a way of ensuring that all the targets were equally memorable, participants were asked to identify each of the targets and verbally recall specific details of the target's story.

### fMRI experiment

#### Stimuli

**Figure 1:**
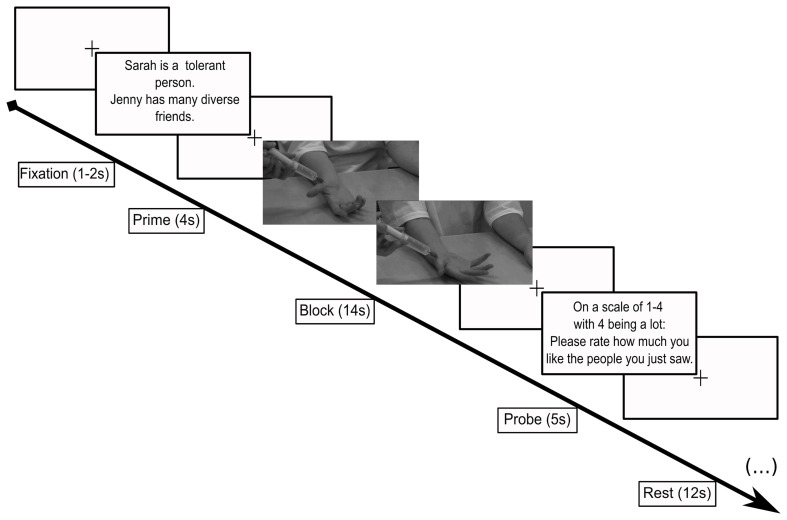
**Scanner paradigm**. Subjects viewed a cue screen reminding them which targets they were about to be see (4 s), followed by a video (14 s) showing the face of the target then zooming to the target's right hand as it received either an injection from a syringe (pain) or a touch from a q-tip (control). Following this condition, subjects were asked to rate how much they liked the people they just saw (5 s). Each of these sequences was followed by a 12 s rest. Each different screen was separated by a jittered fixation screen (1–2 s).

Inside the scanner, participants viewed 7-s movies of each of the targets receiving an injection to the palm of the hand. Stimuli were presented using Psychtoolbox (Brainard, 1997) through MATLAB (v2007a; MathWorks) and projected onto a screen that subjects viewed through a mirror inside the scanner. Each movie clip contained a 1-s frame to show the identity of the target followed by the camera panning down to the target's hand to view the injection. In the control condition, a q-tip replaced the syringe. We used a block design where each block consisted of two trials featuring two likable targets or two disliked targets, either with the q-tip or the syringe, respectively. A block design was used to maximize the detection of differences between conditions (Huettel et al., 2004). The block design was also used in order to build and sustain emotional feelings rather than to ask the subject to quickly move from one emotional state to another, as in event related designs. Indeed, a previous study indicated that it takes about 6 s to the peak of feeling of empathy to physical pain (Immordino-Yang et al., 2009). Trial blocks were preceded by a two-sentence cue (4 s long) to notify the participant as to which targets they were about to view (e.g., In the case of likable targets: “Sarah is a musician in New York.”; “Jill wanted to raise her son to have an open mind.” In the case of hateful targets, “Scott has always believed in white pride; Matt's parents raised him to tolerate others, yet he became racist.”). The cue screen was followed by a fixation cross (1–2 s long), after which the block of movies played for 14-s. The 14-s movie block was filled with two videos of two targets receiving the stimulus (q-tip or syringe) played in sequence with no gap in between. The movie block was followed by a probe screen asking the participants to rate, on a scale of 1–4, how much they liked the targets (5 s). The probe condition was followed by a 12-s rest period. Each functional run consisted of eight blocks total (see Figure 1 for an illustration). Three runs were conducted. The presentation order of the block conditions was performed in a pseudo-random, counterbalanced order to control for 1-back presentation history (Immordino-Yang et al., 2009). Participants were instructed to watch the stimuli and to pay attention to how they felt about the target receiving the touch from the q-tip or the syringe.

#### Image acquisition and preprocessing

Functional and structural Magnetic Resonance Imaging (MRI) was employed using a Siemens 3T scanner. Three functional runs, one anatomical MPRAGE, and one T2 weighted image was acquired for each subject.

Prior to performing the functional scans, structural images were collected in radiological convention with 176 slices, dimensions: 224 × 256 × 176 and then resampled with voxel dimensions 1 × 1 × 1 mm, TR 1950 ms. For functional scans, 173 volumes were acquired, with 37 slices per volume. The TR used was 2000 ms, with an interslice time of 54 and a TE of 30 ms. Inplane resolution was 64 × 64. Voxel resolution was 3.5 × 3.5 × 3.5 mm, with no slice gap, the flip angle was 90°.

All fMRI and structural MRI pre-processing was completed using BrainVoyager (Goebel et al., 2006; Brain Innovation, Maastricht, The Netherlands). Anatomical images were normalized to standard space with the following steps: inhomogeneity correction, alignmment to ACPC space, and then conformation to Talairach space (Talairach, 1988). The fMRI data were first preprocessed for slice-scan time correction using cubic spline interpolation in ascending, interleaved order, after which 3D motion correction was performed along six axes. Functional data were resampled to a voxel-wise resolution of 3 × 3 × 3 mm. The second run of the session was manually coregistered to the MPRAGE anatomical volume and transformed into Talairach space. After motion correction, the runs were aligned to the second functional run from the session. The data were then smoothed with an 8 mm FWHM 3d Gaussian kernel and temporally filtered using a high-pass filter.

#### fMRI analyses

All fMRI analyses were completed using BrainVoyager (Goebel et al., 2006) and MATLAB (version 2007a, Natick, Massachusetts). At the first level of analysis, a general linear model (GLM) was applied using the canonical hemodynamic response function (HRF). Eight explanatory variables were included in the model: fixation, prime, likable targets in pain, hateful targets in pain, likable target q-tip, hateful target q-tip, probe, and rest. Minor head movements along six axes that took place during the runs were included as regressors of no interest into the design matrix to reduce motion artifacts. At the second level of analysis, the individual runs were included in a random effects (RFX) GLM analysis using both a region of interest (ROI) and a whole brain analysis, respectively.

#### Region of interest analyses

To determine differences in activity level and pattern between conditions, ROIs for the a priori regions were hand-drawn on each participant's Talairach transformed anatomical image. ROIs were hand-drawn for each subject based upon anatomical boundaries detailed in Damasio (2005). For the somatosensory cortices, where there is no clear boundary between S1 and S2, boundaries followed those described in Meyer et al. (2011). The ROIs drawn for the striatum were designed to include the caudate, putamen, and nucleus accumbens. We included all the subregions of the striatum because we were interested in general reward processing, as opposed to dissociating specific roles of the components of the striatum (O'Doherty et al., 2004). The regions were drawn on a continuous slice-by-slice basis on each subject's anatomical images using BrainVoyager's hand drawing tool (see **Supplementary Information** Figure 2 for an example labeled subject's brain and the anatomical boundary limits for each ROI).

ROI analysis took the form of a random-effects analysis of the baseline corrected beta values contained in the individual participants' ROIs for each condition. The beta values were entered into two-tailed paired sample *t*-tests for comparison between pairs of conditions (e.g., Pain-Hateful vs. Pain-Likable). To test for a main effect of pain, a Two-Way ANOVA with a factor of liking and hating and the level of pain or no pain was performed. All results are reported at a statistical threshold of *p* < 0.05. The beta values for each ROI for each condition from each participant was also used to analyze correlations with participants' ratings of how much they liked the targets during the scan. These comparisons are reported as significant below the threshold of *p* < 0.05.

The activity in ROIs involved in pain processing (the insula, ACC, and somatosensory cortices) were also analyzed in terms of percent signal change using event related averaging in a *post-hoc* analysis that took place after the data were analyzed using the traditional GLM analysis. This analysis was performed in order to examine potential differences in the time-course for each condition, as well as to better examine the overall direction of the differences between viewing likable people in pain compared with hateful people in pain. Activity in each ROI, as measured in baseline-corrected percent signal change over time, was extracted for each of the conditions in each participant using BrainVoyager. For each ROI, each time point's value was averaged across all participants and plotted as an average time series. Each condition's time series was based upon raw percent signal change relative to the beginning of the trial, which is why each time course is at zero at the beginning of each block (which is time point zero in Figure 3). The statistical analyses of these data took the form of extracting the peak value from the ROI's time course after the second TR of each condition for each subject and performing a Two-Way repeated measures ANOVA on these values with a statistical threshold of *p* < 0.05. The data were analyzed using two levels: likable and hateful, and two factors: pain and no pain, to investigate a main effect of danger or pain, respectively. The first two TRs are excluded to account for hemodynamic delay. The peak activity value, as opposed to the average of the full time series, was analyzed based upon previous studies examining fMRI signal timecourse data (Aziz-Zadeh et al., 2009).

#### Whole brain analyses

For the contrast of seeing hateful people in pain compared with seeing likable people in pain (Pain-Hateful > Pain-Likable), significance is reported in voxels active at false discovery rate (FDR) of *p* < 0.05. For contrasts of seeing the targets undergo pain stimuli compared with the control stimuli, we employed a value *p* < 0.005, corrected for multiple comparison using Monte Carlo cluster threshold estimation running 1000 iterations. This second, lower threshold for regions thought a priori to be involved in pain processing is consistent with recently published results (Decety et al., 2009). The lower threshold also allows for the finding that non-noxious stimuli, such as our control stimuli, can also elicit activity in the pain matrix, which will decrease the contrast between the two conditions (Mouraux et al., 2011).

Participants were asked to rate how much they liked the targets after each block. These ratings were analyzed by taking the mean of each condition's rating for each participant. These ratings were then mean corrected by averaging the group mean and subtracting the group mean rating value for each condition from the individual participant's ratings. These ratings were correlated with beta values from the hand-drawn ROIs for viewing (1) hateful targets in pain and (2) likable targets in pain. These correlations were determined to be significant at the statistical threshold of *p* < 0.05.

#### Psychophysiological interaction (PPI)

A functional connectivity analysis was conducted to determine the interaction between physiological data in terms of the psychological processes of the experiment (Friston et al., 1997). We created 10 mm cubic ROI clusters in the left ACC, and left and right anterior insula by selecting peak voxel activity elicited by the group level whole brain contrast for viewing likable targets in pain subtracted from viewing hateful targets in pain. This ROI selection criterion is similar to recent studies of social pain (Meyer et al., 2013). The same ROI coordinates served as the seed in each subject's data. PPI methodology followed the steps first described in Friston et al. (1997) and was implemented using MATLAB scripts employing functions from BrainVoyager's BVQXtools. At the first level, for each subject, the coordinates of the seed region were used to extract the time series data within the seed. For the analysis, A design matrix was constructed using four predictors consisting of: (1) the boxcar function of the psychological manipulation, (2) the raw signal time course from the seed region for that subject, (3) the interaction between the boxcar function and the raw signal time course, and (4) a confound predictor. The raw time course extracted from the seed was z-transformed and scaled to a minimum value of zero. The boxcar function was created as the difference between the pain-hateful and pain-likable conditions by subtracting the pain-likable boxcar function from the pain-hateful function. This created a box car predictor function for each run consisting of 1′ s for the pain-hateful condition, −1′ s for the pain-likable condition, and 0′ s for the remaining conditions; this was done because we were only interested in the level of likability during pain. Next, the interaction vector was created by multiplying the transformed time series data by the new boxcar function. This third vector was then used as the term by which all other voxel's activity is compared—voxels sharing a similar activity pattern will reveal the “functional connections” to activity in the seed region (Friston et al., 1997). At the second level, the design matrices from the first level were entered into an RFX GLM using a *z*-transformation, corrected for serial correlations. Results were thresholded at *p* < 0.005 corrected using Monte Carlo cluster threshold correction completed over 1000 iterations, based on past studies of empathy that used PPI analyses (Zaki et al., 2007; Benuzzi et al., 2009).

## Results

### Behavioral data

The results from the liking survey administered after each story confirmed that our manipulation of likability was successful; neo-Nazi targets were rated as far less likable than the non-Neo-Nazi targets [mean liked score = 29.3, *SD* = 3.47, cronbach's alpha: 0.69; mean disliked score = 9.9, *SD* = 2.22, cronbach's alpha: 0.79; *t*_(15)_ = 19.88, *p* < 0.00001]. On the open-ended questions, participants most commonly described the hateful targets as hateful, ignorant, unlikable, and racist; participants most commonly described the likable targets as likable, open-minded, fun to hang out with, and interesting. While the subjects were being scanned, after each block, we also asked how much they liked the people in the videos. In these ratings from inside the scanner, Neo-Nazi targets were rated as significantly less liked by our subjects than the non-Neo-Nazi targets [mean liked score = 3.32, *SD* = 0.55; mean disliked score = 1.27, *SD* = 0.35; *t*_(15)_ = 11.94, *p* < 0.00001]. After the initial training session, participants were asked to identify the target from a profile picture and to recall specific details about the target's background. This was a test to ensure that each target was remembered in such a way that he or she could be easily recognized in the scanner. Subjects provided the correct identification of the target 100% of the time and all were able to recall specific details from each target's biography.

### fMRI data

#### ROI analyses

**Figure 2:**
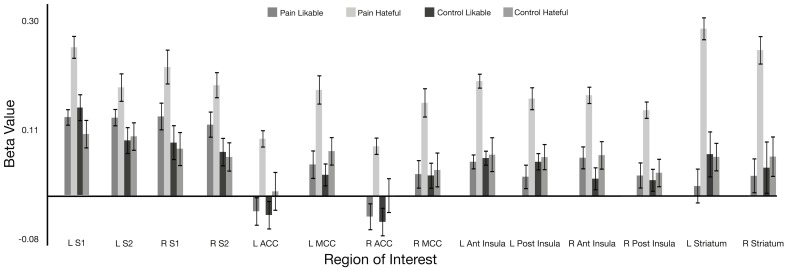
**Beta value for each condition within hand-drawn ROIs in the pain matrix regions**. All ROIs show a significant difference between viewing likable and hateful people in pain at *p* < 0.05. Abbreviations: ACC, anterior cingulate cortex; MCC, middle cingulate cortex; S1, primary somatosensory cortex; S2, secondary somatosensory cortex.

For the comparison of beta values from activity during observation of hateful targets in pain compared with hateful targets in the control condition, the beta values were significantly greater for the pain condition in the left and right ACC, left and right MCC, left and right anterior insula, left and right posterior insula, left and right S1 and S2 and in the left and right striatum. For the comparison between observing likable targets in pain with likable targets in the control condition, no significant differences were found in any of the ROIs. In the comparison of observing the hateful targets in pain compared with observing the likable targets in pain, values were significantly greater for hateful targets in the following regions: left and right anterior insula, left and right ACC, left and right MCC, left PCC, left and right posterior insula, left and right S1, and left and right striatum. There were no significant differences for the opposite contrast. There were no significant differences for comparing the likable and hateful targets in the control condition (for summary of ROI results see Figure 2 and Table 1).

**Table 1:** **Results from hand drawn VOIs in the pain matrix based upon comparison between viewing likable with hateful people in pain**.

**ROI**	**Laterality**	***T*-score**	***P*-value**
ACC	Left	3.727	0.002
MCC	Left	2.723	0.015
ACC	Right	3.804	0.0017
MCC	Right	2.487	0.025
Anterior insula	Left	2.991	0.0091
Posterior insula	Left	2.353	0.032
Anterior insula	Right	2.417	0.028
Posterior insula	Right	2.303	0.036
S1	Left	2.699	0.016
S2	Left	1.569	0.137
S1	Right	2.146	0.048
S2	Right	1.387	0.187
Striatum	Left	3.465	0.003
Striatum	Right	3.209	0.006

Abbreviations: ACC, anterior cingulate cortex; MCC, middle cingulate cortex; S1, primary somatosensory cortex; S2, secondary somatosensory cortex.

The participants' within scan ratings were correlated with beta values from the ROIs for viewing the conditions of hateful targets in pain and likable targets in pain, respectively. This analysis allowed us to determine the effect of individual differences in how the targets were perceived overall on brain activity. Correlating the ratings for likable targets in pain to brain activity while watching likable targets in pain elicited no significant correlations. Correlating the ratings for hateful targets in pain to brain activity for watching hateful targets in pain revealed significant negative correlations (i.e., greater beta values associated with greater dislike of the targets) in left and right MCC [left: *r*_(14)_ = −0.582; *p* = 0.018; right: *r*_(14)_ = −0.523; *p* = 0.037], left and right anterior insula [left: *r*_(14)_ = −0.529; *p* = 0.035; right: *r*_(14)_ = −0.563; *p* = 0.023], left and right S1 [left: *r*_(14)_ = −0.617; *p* = 0.011; right: *r*_(14)_ = −0.544; *p* = 0.029], and left and right S2 [left: *r*_(14)_ = −0.718; *p* = 0.002; right: *r*_(14)_ = −0.611; *p* = 0.012].

**Figure 3:**
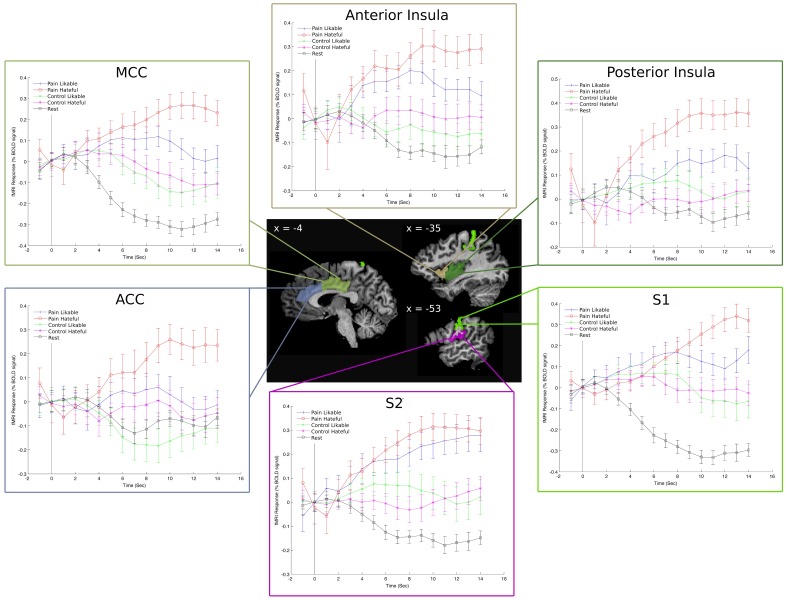
**Event Related Averages for the percent signal change in each condition in left-hemisphere hand-drawn ROIs**. The “rest” line refers to the time course of activity during the rest condition and is there as a comparison to the video-based stimuli. These plots reveal a clear separation among the five conditions throughout the time course of the stimulus presentation and show that the overall result of greater activity for viewing hateful targets in pain is consistent throughout the time course.

We also performed a *post-hoc* analysis of the time series based event related averages for each of the conditions using percent signal change as the dependent measure. The analysis involved comparing the peak values for each condition in each subject's data to statistically compare the differences in percent signal change between conditions. A main effect of pain was found in each ROI, whereas there were no main effects of liking. This is because the viewing likable and hateful targets in the control condition shared roughly equal means, thus diminishing the overall effect of liking. The overall direction of the effect again showed greater BOLD signal change peak values for viewing hateful targets in pain compared with likable targets in pain, however, we note that analyzing the percent signal change over time is fundamentally different from comparing ROI beta values. For summary of *F-* and *p*-values for each ROI, please see Table 2 and Figure 3.

**Table 2:** **Event related averaging results from ANOVA comparisons among conditions for each hand drawn ROI using the peak percent signal change from each condition's timecourse**.

**ROI**	**Laterality**	**Like vs. dislike**		**Pain vs. control**	
		***F*-Value**	***P*-Value**	***F*-Value**	***P*-Value**
ACC	Left	1.562	0.23	5.586	0.032
ACC	Right	1.495	0.24	7.492	0.015
MCC	Left	0.981	0.338	11.029	0.005
MCC	Right	0.416	0.528	13.06	0.003
Anterior insula	Left	1.509	0.238	14.202	0.002
Anterior insula	Right	0.118	0.736	14.523	0.002
Posterior insula	Left	0.818	0.38	9.408	0.008
Posterior insula	Right	0.254	0.622	9.552	0.007
S1	Left	0.11	0.744	5.357	0.035
S1	Right	0.03	0.864	5.454	0.034
S2	Left	0.199	0.662	4.8	0.045
S2	Right	0.14	0.713	5.065	0.04

Abbreviations: ACC, anterior cingulate cortex; MCC, middle cingulate cortex; S1, primary somatosensory cortex; S2, secondary somatosensory cortex.

#### Whole brain analyses

When comparing hateful targets in the pain condition with hateful targets in the control condition, we found increased activity in the right anterior insula, bilateral posterior orbital gyrus (pOrbG), bilateral inferior frontal gyrus (IFG), left superior frontal gyrus (SFG), bilateral dorsal premotor cortex, left paracentral gyrus (paraCG), bilateral precuneus, bilateral inferior parietal lobule (IPL), right anterior middle temporal gyrus (MTG), bilateral extrastriate body area (EBA), right temporal occipital gyrus (TOG), left anterior internal capsule, left thalamus, and right cerebellum. When comparing likable targets in the pain condition compared with likable targets in the control condition, there was a significant increase in the right IPL and the right EBA.

**Figure 4:**
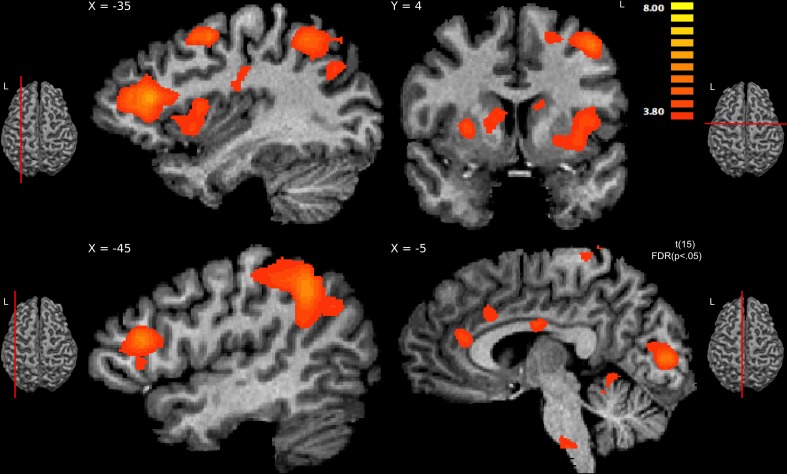
**Regions showing significant activity for viewing hateful people in pain compared with likable people in pain, thresholded at *p* < 0.05, FDR corrected**. The sagittal slice (*x* = −35) shows activity in left hemisphere's anterior insula, middle frontal gyrus, posterior parietal, and dorsal frontal cortex; slice *x* = −45 shows activity in the inferior frontal gyrus; the other sagittal slice (*x* = −5) shows activity in the anterior cingulate cortex and brainstem. The coronal slice (*y* = 4) shows activity in the right caudate and putamen, left caudate, left insula, and left dorsal frontal cortex.

Comparing hateful targets with likable targets in the pain condition yielded increased brain activity for viewing hateful targets in: bilateral ACC and bilateral anterior insula as well as left postcentral gyrus (S1). Other regions revealed to be more active given this contrast include: left IFG, bilateral middle frontal gyrus (MFG), bilateral lingual gyrus, left dorsal precentral gyrus, left supramarginal gyrus (SMG), left angular gyrus (AG), pons, bilateral putamen and caudate (dorsal striatum) and right cerebellum (please see Figure 4, Table 3). There were no significant activations for the opposite contrast (likable targets in pain > hateful targets in pain).

**Table 3:** **Brain regions showing a significant difference in processing hateful people in pain to processing likable people in pain (*p* < 0.05, FDR corrected)**.

**Brain region**	**Laterality**	**Peak *X***	**Peak *Y***	**Peak *Z***	***T*-Value**	***P*-value**	***k***
ACC	Right	5	16	33	5.14845	0.000119	2116
ACC	Left	−11	22	33	5.142391	0.00012	2616
Insula	Left	−31	7	0	5.053652	0.000143	1640
Parietal operculum	Left	−31	−14	30	4.662374	0.000307	876
Post-central gyrus	Right	17	−41	69	4.820237	0.000225	924
Post-central gyrus	Left	−16	−38	72	4.763767	0.000251	418
IFG	Left	−40	28	15	7.401194	0.000002	5974
MFG	Right	26	40	24	8.942296	0.000001	3877
SFG	Left	−7	−14	66	4.947862	0.000175	548
Dorsal premotor	Left	−40	4	48	5.67694	0.000044	3076
STG	Left	−58	−8	0	4.361426	0.000558	537
TPJ	Left	−25	−59	48	6.645249	0.000008	15474
V1	Left	−7	−83	6	5.811272	0.000034	2394
Putamen	Left	−29	7	0	4.94458	0.000176	1270
Putamen	Right	26	4	9	4.781725	0.000242	990
Caudate	Left	−13	16	18	4.450977	0.000467	994
Caudate	Right	11	7	12	4.518043	0.000408	1299
Brain stem	Left	−13	−23	−39	5.18176	0.000112	1820
Cerebellum	Left	−10	−47	−9	5.703101	0.000042	762
Cerebellum	Right	8	−47	−24	4.330719	0.000594	6470

Coordinates are defined in Talairach space and are the peak voxel of a cluster. Abbreviations, ACC, anterior cingulate cortex; IFG, inferior frontal gyrus; MFG, middle frontal gyrus; SFG, superior frontal gyrus; TPJ, temporoparietal junction; V1, primary visual cortex.

#### Psychophysiological interaction

**Figure 5:**
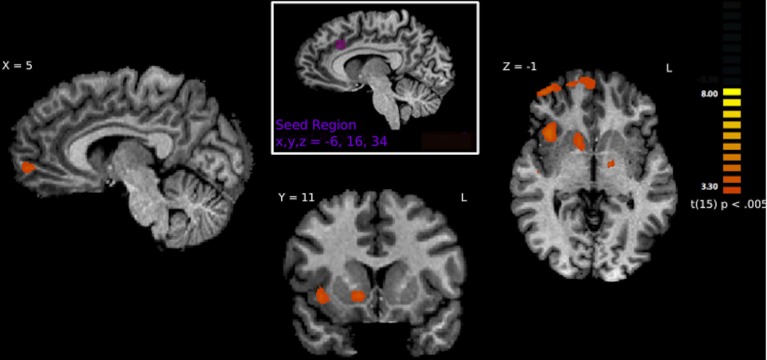
**Brain regions correlated with activity in the left ACC using PPI analysis**. Regions shown represent regions that are more greatly connected to ACC activity while viewing hateful people in pain. Specifically, connections are shown to the right anterior insula, right frontal pole, right nucleus accumbens and right medial prefrontal cortex.

A PPI analysis was used to determine how regions within the pain matrix are functionally connected to other brain regions. We found greater functional connectivity with the left ACC while viewing hateful people in pain compared with likable people in pain in the following regions: right frontal pole, right mPFC, right anterior insula, right frontoparietal operculum, left secondary somatosensory cortex, left superior temporal gyrus, right middle temporal sulcus, left precuneus, left amygdala, left and right putamen, and right nucleus accumbens. Greater functional connectivity while viewing likable people in pain compared with viewing hateful people in pain was found in right lateral occipital cortex (Figure 5). From the right insula seed, connectivity for viewing hateful targets in pain was found to the right and left thalamus, whereas viewing likable targets in pain revealed greater connectivity to the frontal pole, right IFG, left superior posteromedial cortex and right cerebellum. From the left insula seed, greater connectivity to viewing hateful targets in pain was found in the left MTG, and left posterior parietal cortices, whereas viewing likable targets in pain revealed connectivity to the right IFG. For complete summary of the PPI results, see Table 4.

**Table 4:** **Peak voxel Talairach coordinates from regions functionally connected to the ACC during pain processing in likable and hateful targets**.

**Seed**	**Brain region**	**Laterality**	**Peak *X***	**Peak *Y***	**Peak *Z***	***T*-Value**	***P*-value**	***k***
Left ACC	Frontal pole	Right	20	49	3	5.671994	0.000044	2114
	Frontoparietal operculum	Right	44	−8	15	3.992377	0.001177	227
	Anterior insula	Right	38	19	6	5.577087	0.000053	1770
	Superior temporal gyrus	Left	−49	−38	12	4.075235	0.000995	329
	Middle temporal gyrus	Right	41	−20	−6	4.388971	0.000528	404
	Lateral occipital complex	Right	38	−71	−9	−4.548635	0.000384	463
	Precuneus	Left	−16	−50	12	4.809971	0.000229	720
	Amygdala	Left	−25	−5	−6	4.574887	0.000365	546
	Putamen	Left	−13	−8	−3	4.55079	0.000383	327
	Nucleus accumbens	Right	11	10	0	4.267618	0.000674	592
Right AI	Frontal pole	Left	−16	61	12	−4.106384	0.000934	284
	IFG	Right	50	19	9	−5.474448	0.000064	2656
	Superior PMC	Left	−9	−72	45	−3.548958	0.002915	221
	Thalamus	Right & Left	5	−17	−6	5.125062	0.000124	557
	Cerebellum	Right	8	−83	−18	−5.138498	0.000121	521
Left AI	IFG	Left	−43	22	12	−4.541129	0.000390	446
	MTG	Left	−43	−29	6	4.461071	0.000457	1096
	Posterior parietal	Left	−49	−44	42	4.203669	0.000767	353
	Cerebellum	Left	−10	−44	−15	4.374354	0.000544	467

## Discussion

The current study sought to determine how viewing hateful people, compared with likable people, in pain would modulate activity in regions of the brain associated with pain-processing and reward-processing. Based upon previous literature, we predicted: (1) that there would be more activity in components of the pain matrix when subjects observed a likable rather than hateful person in pain; and (2) that regions of the striatum would be more active when viewing hateful individuals receiving a painful injection compared with viewing likable people receiving the same stimulus. In support of the second hypothesis, using hand drawn ROIs and whole-brain analyses, we found increased activity in the striatum for viewing hateful people in pain compared with viewing likable people in pain. However, contrary to our first hypothesis, we found increases in activity in pain-processing regions of the brain when viewing hateful targets experiencing pain. The former findings support the notion of reward-related function associated with witnessing the suffering of a disliked person, i.e., schadenfreude (Cikara et al., 2011). The latter findings reveal that activity in the regions of the pain matrix are not directly wired to be more active for observing likable people in pain, and may be more apt to become active for stimuli that draw stronger top–down attention (Gu and Han, 2007a,b).

Consistent with our hypotheses and with previous findings, we found increased activity in the striatum when viewing hateful individuals experiencing pain. This finding is in line with previous results showing that the regions of the dorsal and ventral striatum are active when viewing out-group members or cheaters experiencing pain (Singer et al., 2006; Takahashi et al., 2009; Hein et al., 2010; Cikara et al., 2011). These findings are usually interpreted in terms of the rewarding nature of viewing someone earning his comeuppance. In our study, participants in post-scan interviews subjectively reported feeling good about watching the neo-Nazi receive the injection. Brain activity in the regions of the striatum could underlie this rewarding experience when seeing a hateful person in pain. In these data, using the whole brain contrast we do not find activity in the nucleus accumbens itself, instead our activity is largely biased toward the dorsal striatum. The nucleus accumbens is commonly associated with generalized unexpected reward, whereas activity elicited in the dorsal striatum, which includes the caudate and putamen, is more activated in the process of linking a motor action to a reward (O'Doherty et al., 2004). The brain activity elicited in our study related to witnessing a hateful person in pain falls in the caudate and the putamen; which closely overlaps with caudate activity found in a study of watching the failure of opposing sports teams (Cikara et al., 2011). It is worth noting, however, that previous studies also show activity in the ventral and dorsal striatum related to tasks unrelated to a feeling or experience of reward, such as viewing strong vs. weak punishment (Strobel et al., 2011), viewing negative images (Carretie et al., 2009) or even viewing unpleasant vs. pleasant images (Gerdes et al., 2010), and thus caution is warranted in interpreting results that connect striatum activity to the subjective experience of reward.

Counter to our hypothesis, we found greater activity in regions of the pain matrix when participants viewed hateful compared with likable targets in pain. The insula, ACC, and somatosensory cortices are commonly activated when one experiences pain and also when viewing others in pain (Avenanti et al., 2005; Hein and Singer, 2008; Lamm et al., 2010). It is possible that increased activity in these regions may be related to increased salience and relevance of the pain-related stimuli rather than to increased empathy-related processing *per se*. Indeed, processing the pain of one's enemy may be more important than processing the pain of another person with less relevance to the self. This interpretation of the results is consistent with previous studies that noted that activity in regions such as the insula can be attributed to top–down effects related to social cognition and/or saliency processing (Gu and Han, 2007a,b; Rilling et al., 2008; Iannetti and Mouraux, 2011).

Furthermore, other regions that were more active when observing hateful compared with likable targets in pain share characteristics with many of the regions found active in studies exploring emotion regulation (for review, see Ochsner and Gross, 2005). Apart from their role in pain processing, several of the regions we find active, namely the ACC and mPFC have been implicated in tasks where participants are asked to regulate responses to threat-based stimuli (Bantick et al., 2002; Tracey et al., 2002; Bishop et al., 2004a,b). These findings complement the interpretation of the current study's findings: depending on the context, activity in regions associated with pain processing can be modulated by attention and relevance of the stimuli to the subject more so than a drive to share a representation of the target's pain personally.

Bruneau et al. (2012) also did not see an in-group bias when witnessing physical pain in in-group members compared to viewing conflict out-group members in pain. They saw an overall increase in pain matrix activity when comparing physical pain in neutral out-group members compared with in-group members (Bruneau et al., 2012). In a limited way, their data complement ours, as their results show that activity in the pain matrix is not hard coded by our desire to share the pain of a target, or to a target's group identity.

A potential limitation to the current results is the lack of differences in activity in pain processing regions while watching likable targets in pain compared with watching likable targets in the control condition. This limitation is addressed through the event related averaging analysis. In this analysis, it is shown that brain activity in pain processing regions for viewing likable targets in pain is different than for viewing them in the control condition, although the effect size is small and not always significant. The time series data also show that the control condition was typically associated with activity in pain processing regions above the baseline level of activity. The results also showed a main effect of pain overall, thus indicating that the pain condition was overall different from the control condition, but the difference was weaker for viewing likable targets in pain. Another account for the lack of a difference in brain activity for viewing likable targets in pain compared to likable targets in the control condition is that perhaps the participants did not feel a sense of liking for the likable targets but instead held a neutral attitude toward these targets. While it may be that the hateful targets were more engaging, there is no indication from the participants' questionnaires or post-scan interviews that the likable targets were not interesting or that the participants held a neutral attitude toward them. The stories of both types of targets were designed to address this issue; the likable targets compared with hateful targets were similarly remarkable in their own stories and life histories, i.e., to the extent that a hateful target worked to hurt others, a likable target worked to help others. That said, the lack of a difference in brain activity for viewing a likable target in pain compared to viewing the likable target in the control condition limits the extent to which the results identify specific functions of these neural circuits, and thus caution is warranted in interpreting these results.

Outside of the pain processing regions, we also found robust activity in the anterior portion of the MFG, when comparing the observation of pain in hateful rather than likable individuals. This region has been implicated in other pain processing studies as well, particularly in tasks involving top–down modulation while processing pain related stimuli (Gu and Han, 2007a,b). The MFG is part of the lateral prefrontal cortex, a region often shown to play a role in emotion regulation and stimuli re-appraisal (Ochsner and Gross, 2005). It was also found active in a study involving viewing faces of hated people (Zeki and Romaya, 2008). In our data, we would ascribe a similar role to the MFG: that of regulation of the emotions and feelings created while viewing the hateful targets.

We predicted that prefrontal cortical regions would be involved in modulation of pain processing networks by likability of the observed person in pain. The PPI results largely support our predictions: activity in the ACC was highly correlated with activity in the medial prefrontal cortex, indicating that the mPFC may be involved in emotion regulation when witnessing hateful individuals in pain compared to witnessing likable individuals in pain. In a previous study, activity of the pACC during the viewing of painful stimuli was positively correlated with activity in the prefrontal cortex, the mid-cingulate cortex and the insula (Benuzzi et al., 2009). In studies examining pain processing and functional connectivity, connections are commonly found between the ACC and the medial prefrontal cortex, as well as regions associated with somatic and/or pain processing, such as the periaqueductal gray, the insula, and the thalamus, (Zaki et al., 2007; Benuzzi et al., 2009; Yoshino et al., 2010; Lamm et al., 2011). Here we show for the first time that the mPFC may also be involved with up-regulation of pain processing when witnessing hateful people in pain.

PPI analysis also revealed functional connections between the ACC and other emotion related brain regions (the insula and amygdala), as well as the nucleus accumbens, frontoparietal operculum, and putamen and the amygdala. These regions are notably found active while viewing a hated person's face (Zeki and Romaya, 2008), and may indicate the additional network of emotion processing aroused by observing a hated person. Specifically, the regions connected to the ACC are regions typical of emotion regulation and reward. Given that the ACC is known to be responsible for pain, cognitive control, and negative affect (Shackman et al., 2011), our findings linking activity in the ACC to the other regions highlight its role as a potential site of integration of the rewarding aspect of the stimulus (the striatum), the monitoring of the interoceptive information (the insula) and the active process of evaluating and regulating ones response (the mPFC).

The results of the present study are novel in that they show brain activity in both the striatum and in the pain matrix regions while viewing hateful people in pain compared with likable people in pain. To our knowledge, no previous studies have presented participants with situations that simultaneously produce increased brain activity in the networks associated with reward processing and observation of pain in others. In this way, our results reveal a novel flexibility in these brain networks. In so doing, these results support the need for future research that can further parse the function of these circuits. In particular, given the flexibility of these regions, research that examines the role of emotion regulation in viewing hateful and likable individuals, such as processes associated with cognitive reappraisal and especially to those processes aimed specifically at predicting an actor's future behavior, will add important understanding to these complex social phenomena.

These results highlight a deep and disquieting aspect of the human experience. At the level of the brain, perhaps the areas of the pain matrix may respond to stimuli under various conditions, and may not directly correspond to the level of liking for a target that a subject may feel while perceiving another person in distress. At the level of society, we see evidence supporting the notion that viewing threatening, hateful people in pain elicits elevated attention to the person in pain in addition to an element of pleasure, which keeps your friend's pain close, but your enemy's closer.

### Conflict of interest statement

The authors declare that the research was conducted in the absence of any commercial or financial relationships that could be construed as a potential conflict of interest.

## Supplementary material

Supplementary Figure S1 **Citations for commonly cited roles for regions of interest. (A)** ^1^(Knutson and Cooper, 2005); ^2^(Yeung and Cohen, 2006); ^3^(Shackman et al., 2011). **(B)** ^1^(O'Doherty et al., 2004); ^2^(Jankowski et al., 2009); ^3^(Bartra et al., 2013). **(C)** ^1^(Craig, 2002); ^2^(Critchley et al., 2004); ^3^(Damasio, 1994). **(D)** ^1^(Iannetti and Mouraux, 2010).

Supplementary Figure S2 Example of hand drawn regions of interest on a single brain. Abbreviations: ACC: anterior cingulate cortex; MCC: mid cingulate cortex; AI: anterior insula; PI: posterior insula; S1: primary somatosensory; S2: secondary somatosensory.
