# Supplementary material for: Witnessing hateful people in pain modulates brain activity in regions associated with physical pain and reward
Source: Front Psychol. 2013 Oct 23;4:772. doi: 10.3389/fpsyg.2013.00772 (PMC3805980; doi:10.3389/fpsyg.2013.00772)
Supplement: Supplementary Figure S1 — Citations for commonly cited roles for regions of interest. (A) 1(Knutson and Cooper, 2005); 2(Yeung and Cohen, 2006); 3(Shackman et al., 2011). (B) 1(O'Doherty et al., 2004); 2(Jankowski et al., 2009); 3(Bartra et al., 2013). (C) 1(Craig, 2002); 2(Critchley et al., 2004); 3(Damasio, 1994). (D) 1(Iannetti and Mouraux, 2010). [file 61706__Data_Sheet_1.ZIP › 61706_Fox_DataSheet1/10_3389_fpsyg_2013_00772 _Fox_Data_Sheet_S1.PDF]

VIGNETTE SAMPLES

VIGNETTE 1: LIKABLE TARGET: STEPHANIE

Stephanie Miller was born to a middle class family in Montreal, Canada. Her father was an orchestral musician and her mom was a high school teacher. She grew up with two younger sisters, who she loved but also fought with on a daily basis. She discovered and developed her interest in music as a young child. It was at the age of 6 when she had her first piano classes at her elementary school in Brossard, a southern suburb of Montreal. Her parents were very loving and extremely supportive of her artistic endeavors. Playing the piano became her passion and as she nurtured that talent, she gradually added vocal lessons to her curriculum. Her parents insisted that she play some sort of sport as well, so when she was 10 she began playing soccer. Stephanie was an excellent sportsman and most everyone agreed that she was a very sweet girl, although she was a bit quiet and reserved. She preferred to have a few close friends as opposed to befriending everyone. Just as she was about to start high school, her father was offered a position in an orchestra in Orlando, Florida. Naturally, the move was not as warmly welcomed by the children who were not eager to leave their lives behind. However, once they moved Stephanie and her sisters adjusted to their new lives and began to make new friends. Their parents began trying to settle into their new lifestyle while the children tried to make friends and readjust their lives. Stephanie and her sister adjusted by joining the culture as best they could. They began making trips to the beach frequently and even taking some surfing lessons. Later, it became clear that the move elicited the formation of a new bond in the three girls and they became each other's best friends. Even when they did eventually make their own friends, they still hung out together a lot.

The population of Orlando was very diverse and the girls enjoyed meeting new friends and being introduced to new cultures. Besides exploring the area, they also particularly enjoyed going to their father's shows. Stephanie continued to take piano and vocal lessons all through high school. At age 17, she developed an interest in acting and became a member of a group which performed comedic musicals. Later that year, Stephanie's family received news of a string of unfortunate events regarding their family back in Montreal. They heard of the tragic death of a beloved cousin and the sudden illness of her mother's sister. Stephanie's mother couldn't bear to be away from her sister and so they decided to move back to Montreal. This move was difficult for the girls since they had become accustomed to their daily routines in Florida, but they too were worried for their aunt's health and devastated by the death of their cousin.

Life back in Montreal was dull compared with Orlando, and Stephanie had a hard time adjusting. She soon became restless and not even her piano lessons consoled her. When she was 19, her aunt passed away from lung cancer. Looking for a distraction, at 20 Stephanie enrolled at the Université de Montréal and began studying music. She began dating the friend of a friend who was also a musician. His name was Brice and he was the drummer in a local rock band. She and Brice decided to tour all of Canada, and so they began taking weekend trips to neighboring cities and regions. During her summers in college, they would take longer trips and eventually even began crossing the border into the United States. When she graduated at 24, she moved to New York City with Brice where she began playing music nightly at a popular piano bar. They moved into a little one bedroom apartment in Brooklyn where she spent her days writing music and exploring New York. Brice was offered a full time job by his friend who owned an art gallery, and he began spending his days there. Stephanie enjoyed attending the art shows that the gallery often hosted.

At this time, Stephanie befriended one of her elderly neighbors. She had noticed the little old man taking walks by himself in the neighborhood with his cane every afternoon. One afternoon she had gone to help him up the steps, and ended up having tea with him and talking for hours. His name was Harry Lowenstein. She was so intrigued by his life story that she found herself not being able to get enough of his stories. Soon, she began taking walks with him. He told her how he was born and raised in Germany. When he first met her, he delighted her with stories of his childhood and adolescence in Europe. Later, he moved on to telling of how he ended up moving to America. The hardships that followed his immigration made him a hero in Stephanie's eyes, and they also made for endless afternoon tea talks. Stephanie felt that she could relate to him after her family had moved around during her childhood. As their relationship grew closer, he revealed to her that his wife had not been able to bear children, and so he had no children of his own. His wife had passed away just two years earlier. The two of them got along so well and had formed such a tight friendship that he told Stephanie he thought of her as part of his family. She had such affection for the old man, and naturally told him that she felt the same way. Mr. Lowenstein imparted to Stephanie the importance of seeing the world through different eyes and to be open to new experiences. While Stephanie was tidying up her apartment one day, she received a large package at the door. It turned out to be from Mr. Lowenstein. He had put together all the sheet music from his favorite songs and concertos. What made the package special, however, was that some of the music was originally from the artist or composer who created the piece. Stephanie could not believe it and insisted that she could not accept the gift, but he would hear nothing of it. He said that he loved to hear her play and that she deserved the best. Stephanie, now 26, continues to master the music in the package. Brice and Stephanie would have Harry over for dinner often and they would all listen to her play piano afterwards.

VIGNETTE 2: LIKABLE TARGET: JESSICA

Jessica Richards was born in a small town in Louisiana. She was an only child. She was the social butterfly of the neighborhood, always playing with the neighbor's children, and when she was older, organizing neighborhood gatherings. Her schooling, religious education, employment, and social life were largely in Christian church affiliated schools, churches, and youth groups. Although she did not agree with all the tenets of the group, it was all she had known in her life. Jessica's parents supported her when she joined what are called "Christian Identity" groups, which are known for the beliefs that the races should be separate. When asked, she claimed that her Christian Identity group is a benign organization supporting the interests of white people. She maintained, "It's just being involved in something you really want to do… Just like a big family." Jessica graduated from high school, but had no concrete plans to attend college. When asked, she would express a desire to earn a degree that would enable her to work in childcare or education, but her plans for a future education were complicated by fears of the "indoctrination" she would receive in a secular university. Instead, she worked as a cashier at a local video store. By 18 she was married to a man who worked as a salesman. She continued to be an active member of Christian Identity, and her husband eventually joined the ranks. The other group members described her as bright, energetic, and approachable. One member referred to her as "the glue" of the group, and went on to say that without her leadership and organization they would fall apart. She insisted that her membership in Christian Identity did not mean that she was racially bigoted or even intolerant of other religions or sexual orientations. She claimed

that her best friend in high school was black and that " it's just the person, not the race, not the religion. If I'm gonna like you, I like you for who you are, not what you are."

Despite these assertions, she would gladly talk about her Anti-Semitic beliefs. She would speak of how she believed that the Jews control the world to further their own evil interests and how American bankers and big business have sold out for Jewish money. She spoke of Jewish men as "morally evil sexual harassers and sleazy abortionists who victimize Aryan women." Jessica drew pride from an argument she had with a friend's dad about whether or not the holocaust actually occurred, or was just merely exaggerated. Jessica contended that the reported number of murdered Jews was way too high to be true.

Two years into her marriage when Jessica was 20, she had her first son, who she named Christopher. In contrast to her upbringing, her son attended an ordinary public school. A turning point in Jessica's life came when her son was four years old. She received a phone call from her son's school informing her that Christopher had destroyed another student's art work, as well as hitting the same student and tearing off his yamika (he was Jewish). When asked about his actions, Christopher said, "He's a dirty Jew. His parents steal money from my parents." The Jewish child, between sobs, said, "I just asked to borrow his yellow crayon and then he hit me." This was a rude awakening for Jessica, who suddenly was aware of the influence she was having on her child and of the values she was imparting upon him. She was appalled that her son was repeating her beliefs without having an ounce of understanding of what those words meant. Seeing the hatred exhibited by her baby boy towards another innocent child gave her a whole new perspective on her beliefs. She realized that her son would never have the opportunity to make up his own mind and it became abundantly clear to her that his life was in her hands to shape. She desperately wanted him to be a free person and to have those opportunities that she never had. She questioned her involvement in Christian Identity and eventually withdrew.

She decided that she didn't want her son to grow up with these values that had been passed down to her. The very first thing that her and her husband did was to have their son apologize to the little Jewish boy while they profusely apologized to his parents. She quickly disbanded the weekly "Jewish information" sessions, and after very little deliberation, quit the entire organization. It was as if her entire life was laid out in front of her, and she could see clearly for the first time. She had known these group members her whole life, and suddenly they appeared to be very different to her. She knew she could never relate to them ever again. Her close friends tried very hard to contact her and talk to her about what was going on, but Jessica became very withdrawn. Any time she doubted the actions she was taking, she looked at her son and immediately became reassured that she was doing the right thing. Jessica felt as though she had to start life all over again, but away from everything that had held her back before. Less than a year after the incident at her son's school, she moved her family to a larger, more diverse town where tolerance was the norm, and not the exception.

Now 25, She reads any and all books she could get her hands on about prejudice and how to handle the topic with children. Her husband encouraged her to go to school, so she began at community college, with plans to transfer to a university in time and get a degree in administrative education. She felt an urgent need to steer the youth away from prejudice. She also became an active member of the town counsel and encouraged the town to discourage racism. Jessica was eager to spread her newfound beliefs, but since she had a while to go before she earned her degree, she began offering to give talks of her story to high schools. The high schools, always looking for ways of reaching out to their students, allowed her this forum. She

told the story of her life, as well as those of all the people she had met through the organization. She emphasized themes of tolerance, co-existence, and most importantly questioning everything that you are taught. She raises her son with these beliefs, as well.

VIGNETTE 3: LIKABLE TARGET: WILLIAM

William Mason was born to a working class family in Detroit, Michigan. His family was one of the few white families in a predominantly black neighborhood. His parents were always reminiscing about how the neighborhood had once been all white and how the black folk had come from "God knows where". They could always be heard complaining about the violent, savage nature of the black folk. Besides complaining about the presence of them in his neighborhood, William's father also complained about their presence in the work force. He loved to rant about how those jobs they held belonged to white folk and how the blacks and Jews were conspiring to keep the white man down. William's father worked in an automobile factory in Detroit and his mother was a stay at home mom. He had a younger sister named Isabella, who he adored. William's childhood was for the most part a happy one until around the age of 10 when his parents began having intense fights. It was at this time that the neighborhood parents stopped letting their kids come over to his house. William reported seeing his dad whipping beer bottles and throwing punches at his mother on many an occasion, while accusing her of cheating on him. Oftentimes, he would try to stand up for his mother, but this usually led to his father's anger being turned on him. The worst part for William was seeing his baby sister, who was around six years old at this time, so scared. He would sometimes sneak to her room during the fights and try to distract her by coloring with her or telling her stories. When the fighting got too bad, they would take off from the house. They would go down the street looking for their friends or for something to do. Because the houses were built close together in their neighborhood, the neighbors could hear the shouting. But because they knew his father, many of their friends' parents wouldn't allow their children to hang around them or to bring them over.

He took to being a loner, however, by age 13 that changed. William had found acceptance in a new group of friends. He had become a "skinhead". He quickly began to champion the beliefs of the White Supremacy movement and developed an increasing hatred for those who were trying to keep the white man weak, namely the Jews, whom he believed controlled the government and the media. He and his fellow gang members used to bully other kids from the neighborhood, usually Jews and Blacks. For the most part, William could be found after school in the basement of one of his buddies' houses, commiserating on the plight of the white man, and how the incessant breeding of the "undesirables" would eventually lead to the white man becoming a minority in his own country. Amongst the group, he was liked by everyone and was described as an intelligent, hard working and genial man.

At 18, William had a turning point in his life. One night while hanging out with his friends at a local bar, his group got into an argument with a group of male students from the local university. His friends, who were also members of William's White Supremacist group, had begun harassing the students. The confrontation started when William's friends had realized the group was composed of Jews. They had begun calling them names and hurling insults at them. Words soon turned into a brawl, with even the bouncers of the bar getting involved. William, with images of prison flashing across his mind, tried his best to hold his friends back and initiate a peace, but he was unsuccessful. One of his friends was hospitalized with a serious head injury, and one of the Jewish students was killed in the incident. William himself was hospitalized for internal bleeding after he received a sharp blow to his stomach.

While recovering in the hospital, he re-evaluated his beliefs and questioned his involvement with the White Supremacist group. He heard that the other Jewish kids involved in the scuffle were devastated over the loss of their friend and that they greatly regretted having ever gone out that night. William himself was greatly perturbed by the death of the Jewish student, and couldn't seem to forgive himself for being involved. He was also haunted by the example that he was setting for his little sister. Recently, he had heard her repeating some of the racist things that he and his friends would say. It hadn't bothered him before, but now he understood the influence that he had on her. He would not want her to go through something like what he just went through, and it suddenly occurred to him that he should set a better example for her, and live a better life for himself. Upon his release from the hospital he drastically changed his life. He stopped hanging out with his old friends. He reunited with his family and began spending time with his mom and sister. His mom pointed out that now since he had more free time, having graduated high school and given up the white supremacist group, he should begin looking for a job. William didn't want a regular job; he wanted to do something that he enjoyed and something that could possibly make a difference in the community. He desperately wanted to help misguided teens steer clear of falling into the same trap he fell into, which was looking for acceptance in the wrong places. He could never forget the Jewish boy who passed away in the brawl, and he still felt like he owed something to the kid. The right job presented itself as a position as a coach at the local YMCA. William had always been an excellent hockey player, and now he could coach the local kids. After five years of coaching, William was promoted to program director of the sports teams. During his years as a coach, he had noticed how sportsmanship overcame most societal boundaries, such as race, socioeconomic status, and so on. The team and the game were everyone's first priorities, and every other concern fell to the wayside. William eventually instituted a local hockey program that would encourage inter-racial interactions that the kids might not get anywhere else. He named the program "Harmony Through Hockey," and it succeeded in bringing children of all races together to play hockey in the Detroit area. The program became the joy of his life since it allowed him to spend time coaching his favorite sport to children while helping them look past racial lines.

VIGNETTE 4: LIKABLE TARGET: KEVIN

Kevin Douglas was born in South Orange, New Jersey. When he was two years old, his father got a new job in California, so the family relocated to a suburb of San Diego. His mother worked as a secretary and his father as an architect. They lived in a middle class suburban neighborhood. Kevin had two older siblings: a sister, Claire, and a brother, Randy. He was a very energetic child and enjoyed being outdoors more than anything. He spent most of his time skateboarding and participating in various little league sports. His mom encouraged him to direct his energy into positive and constructive activities. The age difference between Kevin and his older siblings kept them from playing together too much. When Kevin was ten years old, he befriended the new kid on the block, Jacob. Jacob had just moved from Turkey and had found it hard to adjust to his new life and to make friends in his new neighborhood. Jacob's parents viewed Kevin as a good influence on their son's life. Kevin immediately took a liking to Jacob, took him under his wing and the two quickly became inseparable. They spent their high school years honing their skateboarding skills, going to rock concerts, and playing video games. His parents required him to have a part-time job, and so he started working at an ice cream shop at the mall. A party wasn't a party without Kevin and Jacob. They maintained their friendship throughout high school and still to this day.

Among Kevin's other interests was watching the news on a daily basis. He thought it was very important to stay informed on the world's events, and so he tried to keep up with the news. His parents couldn't understand why anyone would want to watch the news since it mostly reported on the misfortunes and miseries of others. They felt that you can't help these people, so what's the point in watching these stories that make you depressed over the state of the world. Kevin simply felt that information conveyed through the news could teach people something about how people can or cannot relate to each other. Although his parents never quite agreed with him, they still admired and tried to encourage Kevin's interests.

He ended high school not having the grades needed to go to any university that he wanted to go to, so he ended up going to community college. Many of his friends, including Jacob, did the same thing. While taking classes he worked part time at a bank as a teller. After two years, Kevin transferred to Arizona State University where he decided to study history. Since he didn't know anyone in Arizona and was looking for a community at school, he joined a fraternity and moved into the frat house. He led a somewhat typical college life after this. He also began dating Melody around this time, the girl who would become his college sweetheart. His frat brothers liked him and described him as "a nice guy with his head on straight."

For his third year of college, Kevin studied abroad in Barcelona, Spain. He had never been to Europe and thought it would be a great experience, as well as a great opportunity to get away from Arizona. He chose Spain because he had studied Spanish in high school and somewhat in college, so he knew he would be able to get by with the language comfortably in the country. He also felt that going abroad was another way to see other perspectives—much the way he liked to keep up on the news. Kevin was also excited by the reputation Spain has for being a country that never sleeps. Kevin was not disappointed with his decision to go there. He was swept up by every aspect of the Spanish culture, from its siestas and nightlife to its culinary treats. He befriended the other students on the program and took trips with them every weekend to neighboring European countries, such as England, France, Poland, Portugal, and the Netherlands. The program that he was enrolled in set all the students up with a mentor. The mentors were Arizona State University alumni who were now living in Barcelona. Kevin would meet with his mentor, Mike Pendler, about once a month. Mike was a businessman in his thirties who had been living in Barcelona for four years. Mike took Kevin to all his favorite restaurants in Barcelona, and gave him many great tips on local things that Kevin and his friends could do. Since Mike traveled a lot for business, he was also able to give him advice on where to go and what to do when Kevin visited other countries. The two had very similar personalities and interests and they consequently developed a genuine friendship. When it came time to leave Spain at the end of his third year, Kevin was ready to finish college, but nowhere ready to leave his Spanish life and friends. When he did finally return to the US, he finished his last year early.

Upon graduation, he was offered a corporate job in Los Angeles by Melody's father. Although he was not interested in the field at all, he took the offer up and decided to use the opportunity to save some money. He and Melody got an apartment together in downtown Los Angeles. After working at Melody's father's company for a year and a half, Kevin quit. This was around the time that he and Melody broke up. He moved back in with his parents in San Diego and after a year of soul searching, he finally decided to further his education by joining a Master's program in history at the University of Florida. Right after Kevin received his acceptance to the program, Mike contacted him about a job offer in Spain with his company. Kevin was tempted, but ultimately decided to finish his education before he thought about going abroad again.

VIGNETTE 5: HATEFUL TARGET: SARAH

Sarah was born to a poor family in the rural south. Her parents were divorced at an early age and her mother eventually remarried a wealthier man by the time she was eight years old. Although she could remember well her life before her mom became remarried, most of her most formative memories came from the time after she met her stepfather. Her stepfather, Jim, was a businessman who worked at a manufacturing plant. Jim held many extreme right-wing viewpoints and was open about passing these on to Sarah, who was greatly influenced by them. Jim usually came home with some story about how he'd been slighted by his Jew boss and he wanted nothing more than for his stepdaughter to adopt these viewpoints. Many of Sarah's friends and teachers remarked about how loyal Sarah was to her friends and family. If she had made a friend, regardless of the circumstances, she did whatever she could to stand up for her friend, even if it was detrimental or dangerous to her own wellbeing. In one event, a new girl came to Sarah's elementary school and attempted to sit in one of Sarah's friend's seats. Sarah immediately told the girl to go sit somewhere else, and that she was not welcome there. Sarah fell into the middle as a child and rarely stood out in class. What she thought of school, she had learned from Jim: that education was useless, and it only works for pleasing the affirmative action liberals that were trying to take over the country by running the schools. Overall, Sarah made and lost friends easily—sometimes as a result of her loyalty to Jim. Soon she learned to keep most of her opinions veiled somewhat, as most people did not have the right amount of quote "race awareness" to be able to understand her viewpoints. It was during this point that she began sympathizing and relating to the cause and ethos of the neo-Nazi Skinhead groups that inhabited her town.

Sarah grew into a young woman who had solidified her identity much too early in her life. Even once she had moved out of her childhood home without Jim, her stepfather, she maintained the belief that quote "her race was her nation and her nation was her race." She eventually found a job during the summer before her senior year of high school working as a clerk in an insurance office. Her job allowed her the opportunity to find evidence for her beliefs. If a man named Steinberg was particularly vocal about negotiating his claim in the office, she would cite him as being a quote "typical Jew." If a black man did not know what to do with his claim or policy, she just lumped him as a dumb black man. As such, her beliefs were never really challenged and although she was powerless to act directly on these beliefs during her day job, she found an audience in a local right-wing group that met on a regular basis to quote "help conserve and preserve the white race for our children." Her propensity for extreme loyalty proved useful in the group, as she would often sacrifice her own finance and health to help out "brothers and sisters in need." Her voice in the group became the voice of the white and disadvantaged that rose out of dirt and poverty through race-awareness, citing her poor upbringing that changed to middle class under the guidance of her stepfather. She championed the cause of hard work and the need for society to return to a more industrial economy, citing the better pay available to steel workers versus pizza deliverymen. Her philosophy saw the difference between what she felt was an incompetent biological father who represented the worst of modern America, and a militantly conservative stepfather, whom she felt represented the best of what America could be if it could attain a state of purity.

Sarah continues her job in the insurance company, even though she graduated from high school three years ago, and she continues to successfully hide her white supremacist beliefs. Concurrently, she now has risen to be one of the senior women in her neo-Nazi group, helping to organize marches and sit-ins for the members of the group. Her involvement peaked recently

when she helped fund the legal team that was defending some of her group that was indicted for the killing of a black homeless man. Her rationale for supporting the group was that, quote "Here we have four young, perfectly good white men whose lives are over because one homeless black man lie dead, now you tell me, what's worse? Four lives of good young men or one homeless black?" She continues to believe what she has believed since she was younger, finding new evidence in every day situations. She maintains that one day our society will fall into a race war, with whites as a minority trying to retake what they have given to minorities. In maintaining her own race awareness and racial purity, she believes that she can help the whites win the war and maintain the purity of the Aryan nation for generations to come.

Few 22 year olds have the same dedication of purpose that Sarah does. Her background prepared her to maintain the same beliefs and perspectives that drove her to join those groups in the first place. In a recent exchange with a fellow driver on the freeway, she felt as if she had been cut-off in traffic by an expensive looking car pulling out of a synagogue. Rather than accept it as an accident and move on, she followed the driver into a parking lot, muttering to herself, quote "If this J.A.P. thinks she can take our country and do whatever she wants on the road, I'll have to teach her a lesson." She parked nearby the vehicle while the owner went inside. She emerged from her trunk with a can of spray paint and a skull and swastika stencil and painted the image on the windshield of the car. She purposely ran her car into the other car and sped off before she could be reprimanded. Other leaders in the group praised her "willingness to teach the truth."

VIGNETTE 6: HATEFUL TARGET: MATT

Matt was born into an upper middle class white family in a suburban neighborhood. He is an only child. His parents, both corporate workers, his dad an engineer and his mom a financial analyst, sought to teach him about the value of hard work. His parents were present in his life and tried to raise him to the best of their ability, but they were often working late, trying to save up for their retirement. Matt recalls his childhood with some indifference, though he does respect his parents for their ability to work hard. Overall, his parents worked to give Matt a reasonable message: to treat others the way you wish to be treated and to give people a chance.

Matt adopted this message into his worldview, early in life he treated all his peers with decency, regardless of the color of their skin or where they lived. He had many friends from many backgrounds. He maintained friendships outside of his white peer group; in fact, one of his best friends growing up was a boy from Honduras who learned English with Matt's help. The profile of Matt's friends began to change as he grew up, now hanging out exclusively with kids from his own privileged neighborhood. Matt never became an athlete or a scholar, but nonetheless, he took a lot of pride in his body as he grew up, enjoying the fact that he was taller and better looking than many of his peers. After a while, Matt began to feel that his parents were only there to give him things. He never minded them being away at their jobs, as long as they could make it up to him by providing new clothes and cars and the like. As Matt developed, his life's meaning never became clear, and he became all the more bitter for it. Overall, Matt was an unremarkable adolescent, were it not for his developing temper and willingness to belittle others. As he graduated from high school, he became lost in his own lack of drive and bitterness. He applied to many colleges, but did not get in, Matt said, "Probably due to some affirmative action bullshit." Matt opted to remain at home and attend the local community college.

It was in one of his automotive classes that Matt ran into an old friend from the high school football team, Danny. Danny and Matt returned to many of their old ways, harassing

some of the nerdy kids in their classes, saying things like "Hey, if you were so smart, how'd you end up here?" Danny and Matt did little to plan past their junior college education. Matt remarked that he "might as well just be a mechanic, since all the other jobs have been taken by minorities." Matt's new identity allowed him to feel like he had figured out why the things were going wrong for him. Eventually, he and Danny began going to punk rock concerts where they became inculcated into a local group of neo-Nazis—they called themselves POWAR, which stood for Protectors Of the White Aryan Race. At first, Matt thought they were a little extreme, but as he came to know them and listen to their arguments, he began to turn his view and shift his perceptions to focus on the same lines of reasoning as the POWAR leaders. He felt that most of his problems were due to the quote "Jewish and minority takeover of our country." It all began to make sense to him, college sucked because the professors were quote "liberal Jews spewing their Jewish propaganda;" his recent problems getting ahead in life were because, "America has been taken over and handed to the minorities." He felt that one should never be threatened when walking on a street in America, and that crime was a minority problem. However, nothing upset him more than the idea of minorities and Jews taking white women. American women, he said, needed to be protected from the dirty races, in order to cleanse the bloodlines of America. The problems began to color everything about how Matt saw the world. Soon he shaved his head and became one of the most loyal POWAR supporters. On special nights, POWAR would go on their quote "Security Patrols." Matt and his cohort would get drunk and patrol the streets looking for minorities and especially mixed-race couples. POWAR and its leaders felt that they were doing the job of the minority run police. They were the ones out there protecting the real Americans. Their forefathers died for the country only to have the country taken over by minorities. They would begin the night with the chant: quote "The Skinhead revolution is a violent revolution: Take back our country from the Jews and minorities! Sieg Heil!"

Matt's parents and some of his old friends attempted to intervene. They even brought back Matt's Honduran friend to try and prevent him from going too far. His beliefs, however, had been completely solidified by this point. His response to them was fast and harsh. He said things like: "How can you Jew and Nigger-loving retards come here and tell me that America has not been given away? Don't you see how much better it would be if we could have separation of the races? Meanwhile, I'm the one standing up for the real, white Christian America that my forefathers died for. I'm the one who sees how it really is and hasn't been brainwashed by the Jew media." In a fury, Matt left the intervention and went straight to one of his POWAR hangouts. He recounted the incident to a friend and, in a rage, they went downtown smashed the windows of a Chinese owned store and beat mercilessly one of the Hispanic store clerks. They were never arrested or charged for this crime.

Although Matt's violent role in the group has diminished, his history of violence plagues him, as he is on parole for the beating of a Jewish shop owner whom he attacked as he left his shop (the picture above is taken during his court hearing). This history, however, is something Matt takes pride in and uses it to gain credibility within the group as one of their most faithful converts.

VIGNETTE 7: HATEFUL TARGET: REBECCA

Rebecca's childhood would be deemed blessed by most standards. Rebecca was born into extreme privilege. Her father was a wealthy attorney while her mother stayed at home to tend to the Rebecca and her two younger brothers. Rebecca was a talkative little girl who

enjoyed playing sports and helping out with her brothers. She always did well in school, excelling in math and art. Her father was a rather hands-off parent and her mother did her best to raise the children. Consequently, Rebecca's concept of life became skewed toward her privileged upbringing. Through adolescence, Rebecca retained this view of entitlement. She understood from an early age that some people were born with a lot and others were not. For the most part, her parents tried to impart to her that people were born equal and that discriminating against groups was wrong. Consistently among the favorites of her teachers, Rebecca was able to make friends easily and with whomever she pleased. Her friends were fairly diverse in elementary school, although most of them were wealthy. One of her best friends was the daughter of one of their neighbors, and they were from India.

In middle school, she entered a new school in a different part of town. She didn't immediately fit in and soon her priorities shifted from sports and school to hanging out with a new group of underachieving students. Because she no longer participated in her extracurricular activities, she fell out of favor with some of her friends from her younger childhood. In spite of this, Rebecca maintained that she was still working hard, but what had changed was that she had moved to a new school in a mixed race neighborhood. Her new teachers, she thought, resented her for her background and sought to punish her, to show her what it was like for the rest of the students. Over time, Rebecca came to feel that her upbringing was more a curse than a gift, and that in some ways her parents had not done her a favor by raising her in a wealthy home. It was at this point that Rebecca stagnated, doing as little as she could in school and hanging out with her new friends.

Rebecca's attitude toward her minority classmates became more obvious during an essay that she read aloud during one of her creative writing classes. She detailed her feelings and how she had come to realize that her opportunities to have a happy and sustainable life were limited by the influence of quote "everybody that is not white enough to take pride in their heritage." Her teacher tried to intervene, but many factors prevented her from maintaining an even keel. What she had set in motion on that day seemed to take over for how she perceived everything else in her life. One day when she was late to pick up one of her brother at school, he was pushed and shoved around by some of the black students at his school. She arrived to find him disheveled and sobbing. When she called the police, she became frustrated with the operator's accent on the phone and before she could stop herself, she yelled into the phone, quote "Just get me a white cop that I can understand, you fucking paki! What country are you even from!?" Rebecca adapted the frustration and anger from this incident into her broader world view.

When she was 18, she met a boy, Luke, who agreed with her beliefs and they began attending meetings with local white supremacists. In the group, Rebecca felt a sense of community and understanding for her experiences. She felt that these people had a handle on the truth: that America was being handed to minority groups at the expense of resources and opportunities for the white people who founded the nation in the first place. Her sense of identity began to revolve around the group of skinheads with whom she had become affiliated. She drove them to events and concerts and she helped hold the swastika bearing flag during their meetings. Luke, her boyfriend, played guitar in one of the skinhead bands and she wore their skull and eagle adorned clothing everywhere she went. After shows, they would often go out to some of Luke's uncle's land and shoot at beer cans. Their favorite joke was to say to people, quote "Yeah, we like to go out there and shoot some cans…MexiCANS that is!"

Though her relationship to her guitar-playing boyfriend ended within a year of her joining the group, she has done nothing to waiver her support and enthusiasm for the group. The

leaders of the group cite her as a prime example quote "of what should happen to young women all over the country, they should wake up and fight the Jews and Blacks who are taking over our country."

She is aggressive toward any human she meets whom she thinks may be from a minority group. Her reason for doing this is that she feels she is protecting herself. She says, "Well, blacks and those other mud people are more likely to rape people anyway, so I'm just trying to stay safe." In one episode, while pulling her car out of a parking spot, she swung wide, clipping the knee of a black man loading his groceries into his car. Even before he could finish collapsing to the ground, she sped off in a fury. She often bragged about the instance to her cohort in her Aryan group, quote "Well if I'd stopped he probably would have killed me or tried to offer me drugs or something. I probably did him a favor, as long as he wasn't an athlete, which a lot of them are—only thing they can be good at anyway."

She has thoroughly done away with the desire to go to school, citing the fact that education breeds pride in useless knowledge. She does not work and has no need to do so, since she received a trust fund for graduating from high school. Her life then, is spent reading about the history of the Nazi movement and helping out with the needs of her skinhead group.

VIGNETTE 8: HATEFUL TARGET: SCOTT

Scott was born into a working class family in Indiana. His mother was a clerk at the local courthouse and his father was a heavy machinery operator. Scott was raised with one message while he grew up: that hard work should be rewarded and that those born in America with proper lineage deserved access to good jobs. Scott's parents were from families who had been in America for generations going back to the early 1800's. His grandfather had migrated from Mississippi during the depression and had stayed there. His great-great grandfather had fought on the side of confederates during the Civil War--a fact that was honored by the confederate flags flying in front of the house and on all family vehicles. The confederates, they said, had represented what was good and sustainable about America. Scott and his family thought that the result of the Civil War had weakened America and many of the problems in American society could be traced back to that. Scott inherited these beliefs from his parents. Scott took on the viewpoint and even from an early age, it was clear how Scott felt about non-whites in America. In spite of the fact that his family was from a predominantly white town, Scott quickly became an expert at excluding any other children of minority families at his elementary school. Scott had few aspirations in life, except to become like his parents: a good hard-working white American. Scott and his family held education in very low regard. They felt that education would lead to pretentiousness and elitism, as such, Scott was only required to finish high school and then he was free to learn or practice any trade he saw fit. Scott never struggled in school, but he never applied himself either. His strategy was to do a little as possible to get by. Scott saw school as a waste of time. His teachers begrudged dealing with him, most notably his Biology teacher who grew tired of Scott's refusal to read or study the text books. quote "I ain't no monkey," was his main point of contention with the material.

Outside of school, Scott found a group that helped him solidify many of his beliefs about the world. The group was called NSM, or New Socialist Movement. The NSM reinforced Scott's perceptions of the plight of the white man in America. Scott joined the group as a Junior Member, when he was 15. As a junior member, he was in charge of recruiting others at his high school and attending school board meetings to quote "be a watchdog of the Jews in charge." He adopted many of the hallmarks of his Neo-Nazi group, wearing tight jeans with suspenders and

lace up combat boots. He did not completely shave his head, but instead kept it buzzed closely to his head. During this time, a Pakistani family moved to town and Scott took it upon himself to quote “cleanse” the school of any influence this quote “Paki” boy could have over the school’s generally uniform climate. He would often repeat the words of his NSM leaders, quote “If we don’t start standing up for ourselves now, soon our school will be overrun by people like this Paki piece of shit, and we’ll all be out of work and living in fear of the violence that these minorities will bring to our peaceful town.” Scott made it his personal mission to make sure the boy was never safe at the high school. Scott would paint racial epithets on his locker and one day when he and his friends saw the boy walking home alone from school, they swerved towards him in a pickup truck while Scott threw a beer bottle out of the cab at the boy. The bottle shattered over the boys face, breaking his nose and permanently harming his eyesight. Scott was sentenced to six months in jail and two years of parole. Scott was outraged, arguing, quote “That Jew judge threw the book at me when I’m the one standing up for my country, I’m doing the job that our police officers should be doing.”

Scott now works for a friend of his father’s operating a corn-binder. He has not given up his NSM membership and remains a faithful part of the group. In fact, his stint in juvenile hall and the whole ordeal with the boy from Pakistan only reinforced what he believed about the world: that America was being handed over to the minority groups and it was his duty to stand up for it. When the boy and his family moved out of the town, Scott cited this as one of his, and NSM’s largest victories. In fact, Scott and a fellow NSM member were driving by the boy’s house one day after he got out of Juvenile hall and they noticed a moving truck. They quickly assembled their group and arranged a motorcade of pickup trucks to follow the family to the edge of town, after which they took over a local bar and began to party for the night. “One mud family at a time! One mud family at a time!” they chanted as they smashed beer bottles and let fly with their Nazi salutes. The family was devastated by the treatment they received in Scott’s town. But they had, in fact, left town because the boy’s father had gotten a new job on the east coast. Nevertheless, Scott likes to take some credit for quote “doing his part.”

In a short time, Scott married a girl from his high school at a very young age. They have no children yet, but Scott and his wife regularly attend NSM meetings and plan to raise their “own little Aryan army” of their own. They plan to sing the same song that Scott’s parents sang to him when he was growing up: “A is for Aryan, a noble white person, B is for bloodline, the blood that our forefathers spilled to keep our race pure, C is for Creativity, because white people are creative.” They plan to remain in their small town in Indiana for as long as they can, citing the low population of Jews and minorities. They are often heard saying things like, “Hey, our town might not be entirely pure, but we can still try.”

LIKING SCALE

Respond to the following questions by indicating on a scale of 1 to 5, with a 1 for "Strongly Disagree" to a 5 for "Strongly Agree."

Subject of the story: ____________________

Open-Ended questions:

1. How do you feel about this person? What stands out about the story?

Ratings questions:

1. The subject of this story is a likeable person.

Strongly Disagree				Strongly Agree.
1	2	3	4	5

2. The subject of this story is likely to understand the feelings of others outside his or her closest group of friends.

Strongly Disagree				Strongly Agree.
1	2	3	4	5

3. This subject's beliefs are misguided and misinformed.

Strongly Disagree				Strongly Agree.
1	2	3	4	5

4. I would enjoy spending time with this subject.

Strongly Disagree				Strongly Agree.
1	2	3	4	5

5. I think this person will probably not like me.

Strongly Disagree				Strongly Agree.
1	2	3	4	5

6. When this person is in pain, I feel empathy toward him or her

Strongly Disagree				Strongly Agree.
1	2	3	4	5

7. If I were in pain, this person would feel empathy for me.

Strongly Disagree				Strongly Agree.
1	2	3	4	5

8. This person is a unique individual.

Strongly Disagree				Strongly Agree.
1	2	3	4	5
