# Supplementary figures and images for: Witnessing hateful people in pain modulates brain activity in regions associated with physical pain and reward
Source: Front Psychol. 2013 Oct 23;4:772. doi: 10.3389/fpsyg.2013.00772 (PMC3805980; doi:10.3389/fpsyg.2013.00772)

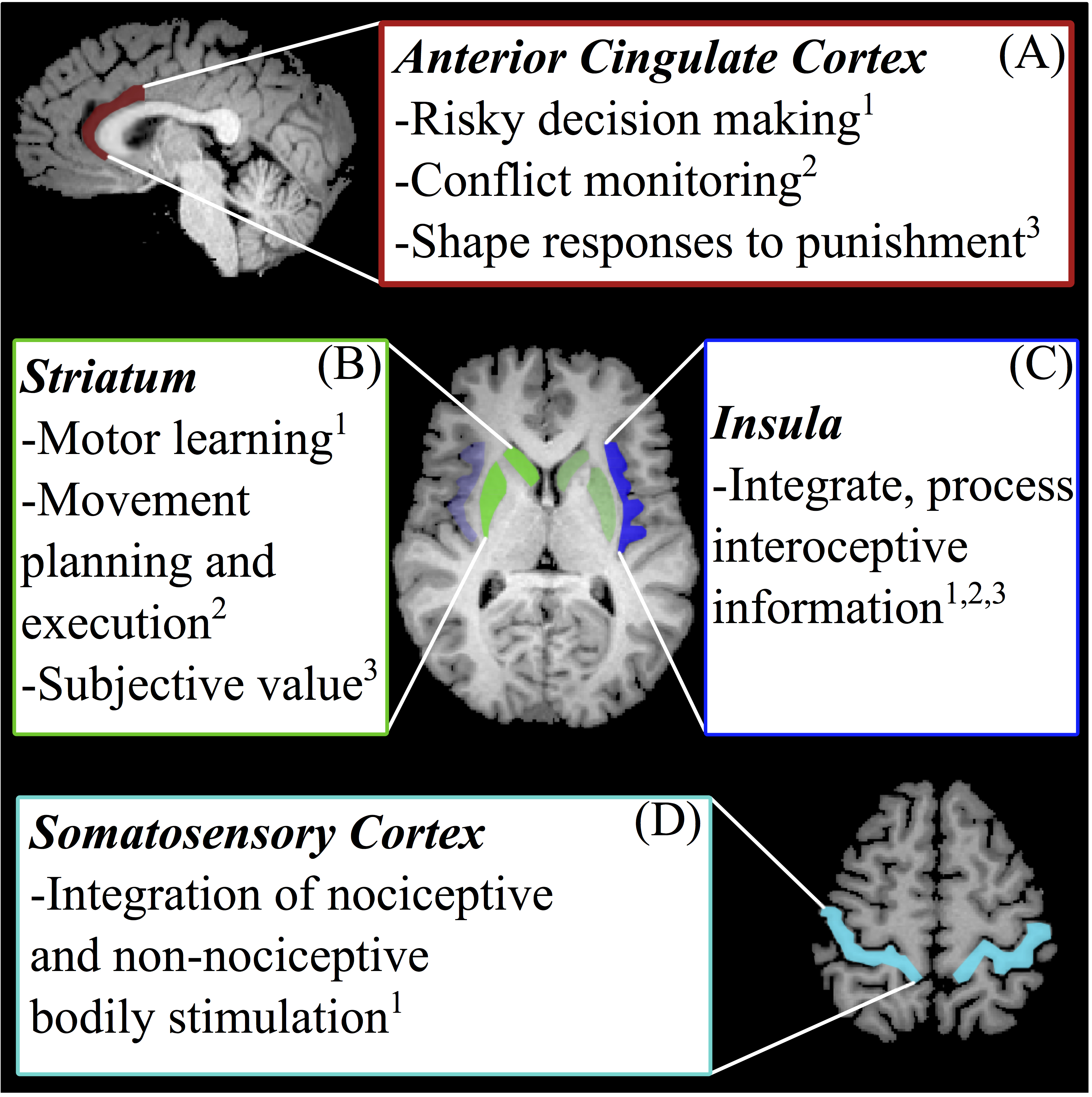

Supplement: Supplementary Figure S1 — Citations for commonly cited roles for regions of interest. (A) 1(Knutson and Cooper, 2005); 2(Yeung and Cohen, 2006); 3(Shackman et al., 2011). (B) 1(O'Doherty et al., 2004); 2(Jankowski et al., 2009); 3(Bartra et al., 2013). (C) 1(Craig, 2002); 2(Critchley et al., 2004); 3(Damasio, 1994). (D) 1(Iannetti and Mouraux, 2010). [file 61706__Data_Sheet_1.ZIP › 61706_Fox_DataSheet1/10_3389_fpsyg_2013_00772 _Fox_Figure_S1.JPEG]

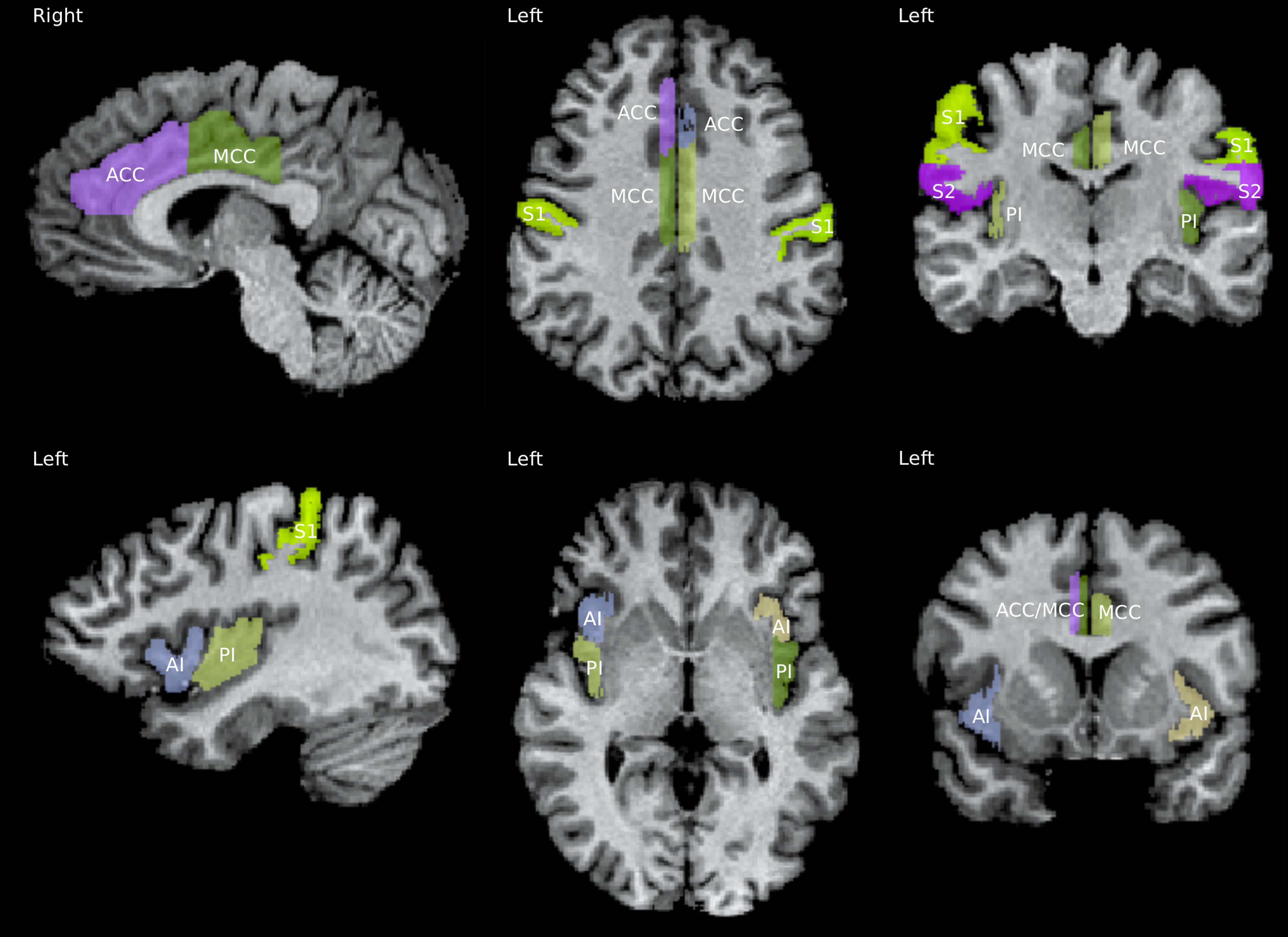

Supplement: Supplementary Figure S1 — Citations for commonly cited roles for regions of interest. (A) 1(Knutson and Cooper, 2005); 2(Yeung and Cohen, 2006); 3(Shackman et al., 2011). (B) 1(O'Doherty et al., 2004); 2(Jankowski et al., 2009); 3(Bartra et al., 2013). (C) 1(Craig, 2002); 2(Critchley et al., 2004); 3(Damasio, 1994). (D) 1(Iannetti and Mouraux, 2010). [file 61706__Data_Sheet_1.ZIP › 61706_Fox_DataSheet1/10_3389_fpsyg_2013_00772 _Fox_Figure_S2.JPEG]
